# Incidence and Risk Factors of Homicide–Suicide in Swiss Households: National Cohort Study

**DOI:** 10.1371/journal.pone.0053714

**Published:** 2013-01-09

**Authors:** Radoslaw Panczak, Marcel Zwahlen, Adrian Spoerri, Kali Tal, Martin Killias, Matthias Egger, for the Swiss National Cohort

**Affiliations:** 1 Institute of Social and Preventive Medicine, University of Bern, Switzerland; 2 Institute of Criminology, University of Zurich, Zurich, Switzerland; Harvard Medical School, United States of America

## Abstract

**Background:**

Homicide–suicides are rare but catastrophic events. This study examined the epidemiology of homicide-suicide in Switzerland.

**Methods:**

The study identified homicide–suicide events 1991–2008 in persons from the same household in the Swiss National Cohort, which links census and mortality records. The analysis examined the association of the risk of dying in a homicide–suicide event with socio-demographic variables, measured at the individual-level, household composition variables and area-level variables. Proportional hazards regression models were calculated for male perpetrators and female victims. Results are presented as age-adjusted hazard ratios (HR) with 95% confidence intervals (95%CI).

**Results:**

The study identified 158 deaths from homicide–suicide events, including 85 murder victims (62 women, 4 men, 19 children and adolescents) and 68 male and 5 female perpetrators. The incidence was 3 events per million households and year. Firearms were the most prominent method for both homicides and suicides. The risk of perpetrating homicide-suicide was higher in divorced than in married men (HR 3.64; 95%CI 1.56–8.49), in foreigners without permanent residency compared to Swiss citizens (HR 3.95; 1.52–10.2), higher in men without religious affiliations than in Catholics (HR 2.23; 1.14–4.36) and higher in crowded households (HR 4.85; 1.72–13.6 comparing ≥2 with <1 persons/room). There was no association with education, occupation or nationality, the number of children, the language region or degree of urbanicity. Associations were similar for female victims.

**Conclusions:**

This national longitudinal study shows that living conditions associated with psychological stress and lower levels of social support are associated with homicide-suicide events in Switzerland.

## Introduction

Violent deaths due to injuries and accidents contribute importantly to premature mortality and life-years lost. Defined as the homicide of one or several individuals followed by suicide of the perpetrator, homicide-suicide events, also referred to as ‘extended suicide’, ‘murder–suicide’ or ‘dyadic death’ represent a small but important segment of injury deaths [1], [2]. Homicide-suicides represent a distinct category of violent deaths with characteristics that distinguish them from both homicides and suicides [1], [3], [4]. Although these events are rare, they have devastating effects on families and communities and usually attract much coverage in the popular media [5], [6].

Homicide-suicide events generally include one perpetrator and one victim: the majority of perpetrators are men and most victims are women [1]. Events often occur in families or within settings of current or former intimate relationships and the use of firearms is common [5]. In the context of intimate partner homicide-suicides the phrase ‘amorous jealousy’ [7] is often used to describe the motives of killers in younger age-groups, and ‘mercy killing–suicide’ [3] to describe the actions of some killers of older or sick women. Recently the broader category of ‘male proprietariness’ has been proposed to cover both instances of intimate partner homicide-suicides [8]. These events have been called ‘extended’ or ‘altruistic’ suicides because the depressed mother tends to perceive the child as exclusively dependent on her and intends to ‘save’ herself and her child or children from an unbearable world [7].

Several authors have argued that the rate of homicide-suicide varies less between countries than the rate of homicide, such that homicide-suicide as a percentage of all homicides is negatively correlated with the rate of homicide [2], [9]. The association between the two rates has recently been re-examined in a systematic review of 49 studies by Large et al [10]. In the United States of America (USA) the rates of homicide-suicide correlated closely with the rates of homicide whereas outside the USA homicide rates were considerably lower but rates of homicide-suicide varied widely [10]. Recent data from South Africa fit the pattern observed in the USA, with high homicide and high homicide-suicide rates [11]. These data support the notion that in settings with high homicide rates measures to reduce these rates would also reduce homicide-suicide [11].

Previous studies of homicide-suicide events were generally based on case series and focused on forensic and criminological aspects of the events [9], [10], [12]–[16]. To our knowledge no data from population-based cohort studies are available. We attempted to fill this gap by identifying homicide–suicide events within households in the Swiss National Cohort (SNC), a longitudinal study of the entire Swiss population [17]–[19].

## Methods

### Study Population

We analysed the data from the SNC, which has been described in detail elsewhere [17] [20]. Briefly, the SNC is a longitudinal mortality study of the whole Swiss population, based on linkage of the 1990 and 2000 census data with the mortality records. Switzerland does not have a system of unique identification of residents which can be used to deterministically link records from different databases. Mortality and emigration records covering the period December 5^th^ 1990 to December 31^st^ 2008 were therefore linked to the census using probabilistic record linkage [17]. All data were provided through contractual agreements with the Swiss Federal Statistical Office. The SNC has ethical approval from the Ethics Committees of the Cantons of Zurich and Bern, which covers the present analyses.

The census consisted of three questionnaires: one for the individual, a household questionnaire and a questionnaire on the building. Each person living in Switzerland at the time of census (or their legal representative) was obliged by law to complete the personal questionnaire. The household questionnaire, which covers the number of persons living in the household, including children and other relatives, is completed by the head of the household. The head of household is defined as the person economically responsible for the household. All questionnaires and a complete list of variables are available at http://www.swissnationalcohort.ch.

### Definition of Homicide-suicide Events

The events of interest were clusters of one or several homicides and one suicide that occurred within a household of at least two persons. We included households of married or cohabitating couples with or without children that were recorded in one or both of the 1990 or 2000 censuses. The analysis excluded individuals living in single person or single parent households, people living in institutions, and households of several adults sharing accommodation, for example students. Causes of death linked to the SNC were coded according to the eighth revision of the International Classification of Diseases, Injuries and Causes of Death (ICD-8) until 1994, and the 10th revision (ICD-10) thereafter. The analysis limited cases to deaths registered with the primary cause as defined by the codes:

- 953–958 for violent suicides; 960–968 for homicide (ICD-8);
- X70– X82 for violent suicides; X91– X99 and Y00– Y09 for homicide (ICD-10).

Deaths had to occur in sequence, with the homicide or homicides occurring before the suicide. A maximum time window of 14 days between the two events was allowed.

### Variables

We used demographic and socio–economic data at the level of the individual, data on the composition of households and crowding, and data on the characteristics of the area. At the individual level nationality was grouped into categories ‘Swiss’, ‘Other European’ and ‘Other or unknown’. Residence status was in four categories: ‘Swiss’, ‘Permanent residence permit’, ‘Annual/seasonal permit’ and ‘Other’. Reported religious affiliation was categorised into four groups: ‘Protestant’, ‘Catholic’, ‘No affiliation’ and ‘Other affiliation/unknown’. ‘No affiliation’ was an explicit response option in the census questionnaire. The analysis considered four categories of educational attainment (‘Compulsory schooling or less’, ‘Secondary’, ‘Tertiary’ and ‘Unknown’). Categories of socio–professional status classification were collapsed into 10 hierarchical groups reflecting the position and status of individuals in the labour market. At the level of the household we considered the number of adults living in the household (2 or >2 adults), the number of children (0, 1, ≥2 children) and the number of persons per room (combining bed and living rooms): <1, 1–<2 and ≥2. Finally, the study considered characteristics of the area of residence. The level of urbanisation was in three categories: ‘Urban, ‘Peri-urban’ and ‘Rural’ according to the classification of communities provided by the Swiss Federal Office of Statistics, and language region in three: ‘German’, ‘French’, and ‘Italian’.

### Statistical Analysis

The analysis used descriptive statistics to characterize the perpetrators and victims of homicide-suicide events. A Cox proportional hazard regression was used to examine associations of socio-demographic characteristics with the hazard of becoming a perpetrator (death by suicide) among the adult male Swiss population living in eligible households. Similarly - the hazard of becoming a victim (death by homicide) was modelled among the adult female Swiss population. The observation time started at the date of the earliest census the individual was recorded as a member of a household with two or more persons (December 4^th^ 1990 or December 5^th^ 2000) and ended at the date of death by homicide or suicide, the date of emigration or December 31^st^ 2008, whichever came first. The study censored the time of observation on December 5^th^ 2000 in individuals who lived in households with two or more persons in 1990 but were recorded as living in ineligible households or institutions in 2000.

Age-adjusted hazard ratios were obtained by using age as the time scale in the Cox proportional hazard models. Results were expressed as hazard ratios (HR) with 95% confidence intervals (CI). We obtained *p* values from likelihood ratio tests. The study used Stata (College Station, Texas, USA, version 11.0) for all analyses.

## Results

### Study Population

The 1990 census included a total of 6,873,687 residents and the 2000 census 7,288,010 residents, for a combined total of 8,527,638 potentially eligible individuals. A total of 1,114,221 deaths were recorded in Switzerland between December 5^th^ 1990 and December 31^st^ 2008. Among these 1,052,619 (94.5%) deaths could be linked to a census record using probabilistic record linkage [17]. The analysis excluded individuals who lived in an ineligible household at both censuses or at one census, if no eligible household was identified in the other census. 1,044,350 individuals living in single person households, 256,915 individuals living in single parent households, 136,460 people living in shared accommodation and 235,574 persons living in institutions were also excluded. A total of 6,854,339 individuals living in 2,466,30 eligible households were included in the analysis. Among these 1,552,863 individuals (from 530,829 eligible households) were recorded in the census 1990; 1,685,404 individuals (from 777,150 eligible households) were recorded in the census 2000 and 3,616,072 individuals (from 1,158,327 eligible households) were recorded in both censuses.

### Homicide–suicide Events

We identified 73 homicide–suicide events involving 158 deaths. In 1991 and 2001, when ascertainment of events was near complete, there were six and seven events, respectively. The homicide-suicide offending rates per 100,000 were 0.09 and 0.10 and victimization rates 0.13 and 0.15 in 1991 and 2001 respectively. This translated into a rate per 1,000,000 eligible households of 3.01 in 1991 and 2.95 in 2001 (*p* = 0.97 for difference). Most events related to husbands killing their wife (55 events; 74.3%) or killing their wife and offspring (six events; 8.2%) (**Table 1**). In five events the perpetrator was a woman: the victims included three husbands, one son and two children. The five women were middle aged (range of 34–51 years), married, Swiss nationals. The perpetrators died by suicide on the day of the homicides in 63 (86.3%) cases. In eight instances (11.0%) the suicide occurred one day after the homicides and in two cases it occurred two and eight days after the homicide. In 67 of the 73 suicides (91.8%) a handgun, rifle or other firearm was used. Similarly, 73 of the 85 cases of homicides (85.9%) involved firearms. Other methods included homicide by strangulation, suffocation and stabbing, and suicide by hanging.

**Table 1:** Relationships between perpetrators and victims in 73 homicide-suicide events, Switzerland 1991–2008.

		Events		Victims		Total deaths	
	Murder victim	No.	%	No.	%	No.	%
***Male perpetrator***							
(n = 68)	Wife	55	75.3	55	64.7	110	69.6
	Wife and adolescent offspring	5	6.8	15	17.6	20	12.7
	Wife and adolescent son	1	1.4	2	2.4	3	1.9
	Adult son	1	1.4	1	1.2	2	1.3
	Adolescent son	2	2.7	2	2.4	4	2.5
	Adolescent daughter	1	1.4	1	1.2	2	1.3
	Other adult male	1	1.4	1	1.2	2	1.3
	Other adult female	2	2.7	2	2.4	4	2.5
***Female perpetrator***							
(n = 5)	Husband	3	4.1	3	3.5	6	3.8
	Adolescent son	1	1.4	1	1.2	2	1.3
	Adolescent offspring	1	1.4	2	2.4	3	1.9
***Total***		73	100	85	100	158	100

### Characteristics of Perpetrators, Victims and the Population at Risk

**Figure 1:**
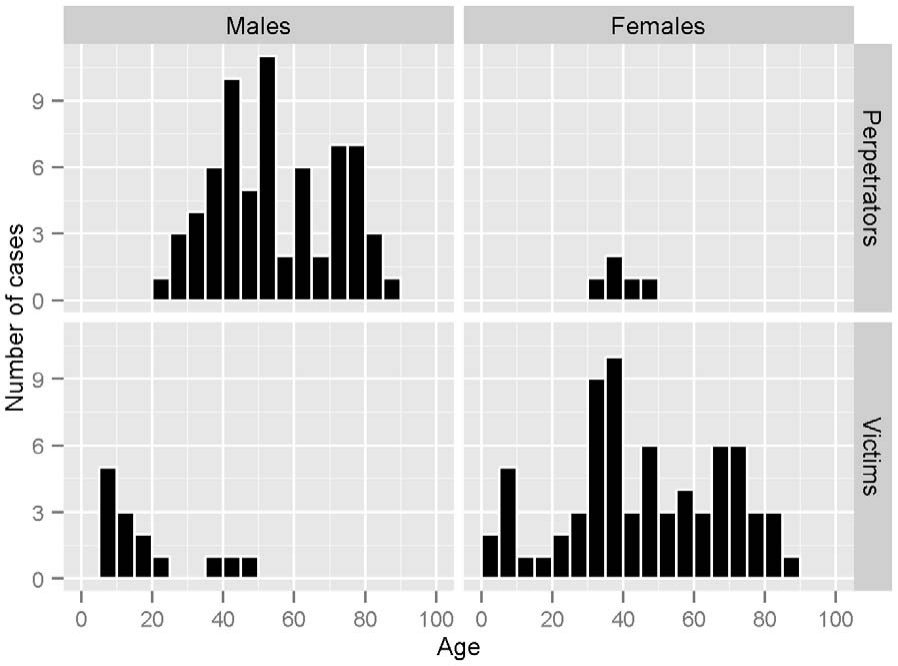
Age distribution of homicide and suicide victims across genders. Homicide–suicide events in Swiss households 1991–2008.

Comparing the socio-demographic characteristics of perpetrators and victims with the population at risk shows marked differences in the sex and age distribution: 68 of the perpetrators (93.2%) were male and 71 (83.5%) of the victims were female (**Table 2**). The age distribution of male perpetrators showed a bimodal pattern, with peaks around 45 years and 75 years (**Figure 1**). Of note, no perpetrator was younger than 20 years. The distribution in female victims showed a trimodal distribution, with peaks around 10 years, 40 years and 75 years of age. Among male victims children and young adults dominated: eight out of 14 (57.1%) male victims were under the age of 18 years.

**Table 2:** Characteristics of perpetrators of homicide-suicides, victims, and source population, Swiss National Cohort 1991–2008.

*Level*		*Characteristic*	*Perpetrators*		*Victims*		*Population at risk*	
			No.	%	No.	%	No.	%
		Total	73	100.0	85	100.0	6,854,339	100.0
***Individual***	***Sex***	Male	68	93.2	14	16.5	3,500,564	51.1
		Female	5	6.8	71	83.5	3,353,775	48.9
	***Age (years)***	0–19	0	0.0	19	22.4	1,713,519	25.0
		20–29	4	5.5	6	7.1	8,48,182	12.4
		30–39	13	17.8	20	23.5	1,085,815	15.8
		40–49	17	23.3	11	12.9	984,770	14.4
		50–59	13	17.8	7	8.2	877,223	12.8
		60–69	8	11.0	9	10.6	641,112	9.4
		70–79	14	19.2	9	10.6	483,609	7.1
		80+	4	5.5	4	4.7	220,109	3.2
	***Marital***	Single	2	2.7	23	27.1	2,657,797	38.8
	***status***	Married	65	89.0	58	68.2	3,991,694	58.2
		Widowed	0	0.0	1	1.2	67,378	1.0
		Divorced	6	8.2	3	3.5	137,470	2.0
	***Nationality***	Switzerland	61	83.6	74	87.1	5,407,953	78.9
		Rest of Europe	12	16.4	11	12.9	1,292,792	18.9
		Other countries/unknown	0	0.0	0	0.0	153,594	2.2
	***Residence***	Swiss	61	83.6	74	87.1	5,407,953	78.9
	***status***	Permanent residence	7	9.6	8	9.4	1,038,269	15.2
		Short-term residence permit	5	6.9	3	3.5	338,723	4.9
		Other	–	–	–	–	69,394	1.0
	***Religion***	Protestants	25	34.2	34	40.0	2,504,114	36.5
		Catholics	28	38.4	33	38.8	3,003,618	43.8
		No religious affiliation	14	19.2	14	16.5	645,216	9.4
		Other/unknown	6	8.2	4	4.7	701,391	10.2
	***Education***	Compulsory education or less	17	23.3	23	27.1	1,692,062	24.7
		Secondary	37	50.7	38	44.7	2,661,270	38.8
		Tertiary	18	24.7	5	5.9	939,798	13.7
		Unknown	1	1.4	3	3.5	157,696	2.3
		School age or younger	0	0.0	16	18.8	1,403,513	20.5
	***Occupation***	Top management	1	1.4	1	1.2	68,046	1.0
		Middle management	2	2.7	2	2.4	261,991	3.8
		Lower management	4	5.5	6	7.1	530,751	7.7
		Independent professions	9	12.3	5	5.9	355,893	5.2
		Skilled non-manual labour	6	8.2	7	8.2	634,239	9.3
		Skilled manual labour	10	13.7	0	0.0	302,654	4.4
		Unskilled manual labour	4	5.5	7	8.2	516,012	7.5
		Not classified	10	13.7	3	3.5	709,298	10.3
		Unemployed	3	4.1	2	2.4	120,631	1.8
		Not in paid employment	24	32.9	36	42.4	1,951,310	28.5
		In compulsory education	0	0.0	16	18.8	1,403,514	20.5
***Household***	***No. of adults***	2	70	95.9	82	96.5	6,414,873	93.6
		>2	3	4.1	3	3.5	439,466	6.4
	***No. of children***	0	37	50.7	37	43.5	2,296,537	33.5
		1	12	16.4	12	14.1	1,391,698	20.3
		> = 2	24	32.9	36	42.4	3,166,104	46.2
	***No. of persons*** ***per room***	<1	44	60.3	51	60.0	4,29,8374	62.7
		1–<2	25	34.2	30	35.3	2,386,802	34.8
		> = 2	4	5.5	4	4.7	169,163	2.5
***Area***	***Language region***	German	54	74.0	62	72.9	4,960,396	72.4
		French	15	20.5	17	20.0	1,599,605	23.3
		Italian	4	5.5	6	7.1	294,338	4.3
	***Urbanization***	Urban	22	30.1	23	27.1	1,758,448	25.7
		Peri-urban	30	41.1	35	41.2	316,4160	46.2
		Rural	21	28.8	27	31.8	1,931,731	28.2

The proportion of married and divorced individuals was higher among both perpetrators and victims compared to the population at risk (**Table 2**). Similarly, individuals with no religious affiliation and people with secondary or tertiary education were more common among perpetrators compared to the population at large. Finally, among occupational categories the self-employed and skilled manual workers were more likely to be perpetrators than individuals in other professional categories. There were few differences in other characteristics, for example language or degree of urbanisation. These comparisons with the population at risk have to be interpreted with caution because of the pronounced differences in the sex distribution.

### Factors Associated with Men Committing Homicide-suicide

The Cox regression analysis was based on 2,624,839 men living in an eligible household, 35.9 million person-years of follow-up, and 68 events of men committing homicide-suicide. The small number of women perpetrators (n = 5) precluded analyses in women. The risk of committing homicide-suicide was increased in 30–49 year olds compared to younger men, and again increased in men aged 70 and above (*p* = 0.03, **Table 3**), thus confirming the bimodal pattern seen in the crude numbers in **Table 2**. The risk was also increased in divorced men living with a new partner, compared to married and single men (*p* = 0.02) and foreigners without permanent residency (*p* = 0.03). The risk was higher in men without religious affiliation compared to Catholics (*p* = 0.02), but the association overall with religion failed to reach conventional levels of statistical significance (*p* = 0.12). Finally, the risk increased with crowding (*p* = 0.01), with an almost five times higher risk in households with two or more persons per room. Of note, there was no evidence for an association with education, occupation, nationality, the presence of children in the household, the language region and the degree of urbanization.

**Table 3:** Individual, household and area characteristics and the risk of committing homicide-suicides in men, Swiss National Cohort 1991–2008.

*Level*	*Variable*	*Categories*	*Hazard ratio*	*(95% CI)*	*p*
***Individual***	***Age (years)***	20–29	0.50	(0.16–1.60)	0.03
		30–39	1.00	–	
		40–49	1.37	(0.62–3.06)	
		50–59	1.25	(0.55–2.86)	
		60–69	1.09	(0.43–2.77)	
		70–79	2.87	(1.27–6.46)	
		80+	2.26	(0.71–7.22)	
	***Marital***	Single	0.43	(0.10–1.88)	0.02
	***status***	Married	1.00	–	
		Widowed	–	–	
		Divorced	3.64	(1.56–8.49)	
	***Nationality***	Switzerland	1.00	–	0.15
		Rest of Europe	1.22	(0.65–2.31)	
		Other countries/unknown	–	–	
	***Residence status***	Swiss	1.00	–	0.03
		Permanent residence permit	0.78	(0.35–1.73)	
		Annual or seasonal residence permit	3.95	(1.52–10.2)	
	***Religion***	Protestants	0.99	(0.56–1.74)	0.12
		Catholics	1.00	–	
		No religious affiliation	2.23	(1.14–4.36)	
		Other/unknown	1.40	(0.57–3.43)	
	***Education***	Compulsory education or less	1.24	(0.68–2.27)	0.84
		Secondary	1.00	–	
		Tertiary	0.90	(0.50–1.62)	
		Unknown	1.18	(0.16–8.68)	
	***Occupation***	Top management	0.72	(0.08–6.45)	0.18
		Middle management	0.39	(0.07–2.16)	
		Lower management	0.49	(0.12–1.97)	
		Independent professions	1.10	(0.33–3.66)	
		Skilled non-manual labour	1.00	–	
		Skilled manual labour	2.15	(0.67–6.84)	
		Unskilled manual labour	1.09	(0.27–4.38)	
		Not classified	1.33	(0.42–4.27)	
		Unemployed	2.53	(0.46–13.8)	
		Not in paid employment	0.73	(0.20–2.63)	
***Household***	***No. of adults***	2	1.00	–	0.74
		>2	0.83	(0.26, 2.64)	
	***No. of children***	0	1.00	–	0.58
		1	0.98	(0.48–2.04)	
		> = 2	1.36	(0.70–2.65)	
	***No. of persons***	<1	1.00	–	0.01
	***per room***	1–<2	2.00	(1.17–3.43)	
		> = 2	4.85	(1.72–13.6)	
***Area***	***Language***	German	1.00	–	0.71
	***region***	French	0.82	(0.45–1.51)	
		Italian	1.26	(0.46–3.50)	
	***Urbanization***	Urban	1.00	–	0.41
		Peri-urban	0.68	(0.39–1.21)	
		Rural	0.86	(0.47–1.58)	

Hazard ratios adjusted for age from Cox regression models based on 2′624′839 men older than 18 years living in eligible households in Switzerland. *p* values from log-likelihood ratio tests. CI; confidence intervals.

### Factors Associated with Women Dying in Homicide-suicide Events

This analysis was based on 2,515,961 women living in eligible households, 35.1 million person-years of follow-up and 62 women killed in homicide-suicide events. The small number of male victims (n = 14) precluded analyses in males. The risk of dying in homicide-suicides was increased in women aged 30–39 years and women aged 80 years and older compared to other women (*p* = 0.05, **Table 4**). Other risk factors mirrored those found in men committing homicide-suicide acts: the risk of being killed was associated with religion (*p* = 0.02), with a substantially increased risk of dying in homicide-suicide events among women with no religious affiliation compared to Catholics. Finally, it was higher in overcrowded households than in less crowded conditions although the association failed to reach statistical significance (*p* = 0.07). Again, there was no evidence for an association with education, occupation, nationality, the presence of children in the household, the language region and the degree of urbanization.

**Table 4:** Individual, household and area characteristics and the risk of dying in homicide-suicides in women, Swiss National Cohort 1991–2008.

*Level*		*Characteristic*	*Hazard ratio*	*(95% CI)*	*p*
***Individual***	***Age (years)***	20–29	0.36	(0.14–0.98)	0.050
		30–39	1.00	–	
		40–49	0.46	(0.21–1.02)	
		50–59	0.41	(0.17–0.97)	
		60–69	0.74	(0.34–1.64)	
		70–79	1.13	(0.51–2.50)	
		80+	1.48	(0.50–4.36)	
	***Marital***	Single	1.03	(0.35–3.10)	0.70
	***status***	Married	1.00	–	
		Widowed	0.63	(0.08–4.69)	
		Divorced	2.04	(0.64–6.57)	
	***Nationality***	Switzerland	1.00	–	0.42
		Rest of Europe	1.04	(0.49–2.21)	
		Other countries/unknown	–	–	
	***Residence status***	Swiss	1.00	–	0.87
		Permanent residence	0.93	(0.39–2.17)	
		Short-term residence permit	1.14	(0.27–4.80)	
	***Religion***	Protestants	1.63	(0.91–2.92)	0.02
		Catholics	1.00	–	
		No religious affiliation	3.29	(1.56–6.95)	
		Other/unknown	0.95	(0.28–3.20)	
	***Education***	Compulsory education or less	0.88	(0.49–1.55)	0.48
		Secondary	1.00	–	
		Tertiary	0.75	(0.29–1.93)	
		Unknown	2.46	(0.75–8.06)	
	***Occupation***	Top management	4.50	(0.55–36.5)	0.17
		Middle management	0.92	(0.11–7.49)	
		Lower management	1.22	(0.36–4.16)	
		Independent professions	2.65	(0.84–8.39)	
		Skilled non-manual labour	1.00	–	
		Skilled manual labour	–	–	
		Unskilled manual labour	1.69	(0.56–5.04)	
		Not classified	0.33	(0.07–1.58)	
		Unemployed	1.89	(0.39–9.08)	
		Not in paid employment	1.17	(0.47–2.93)	
***Household***	***No. of adults***	2	1.00	–	0.71
		>2	0.81	(0.25–2.59)	
	***No. of children***	0	1.00	–	0.59
		1	0.67	(0.30–1.52)	
		> = 2	0.96	(0.48–1.92)	
	***No. of persons*** ***per room***	<1	1.00	–	0.07
		1–<2	1.66	(0.94–2.94)	
		> = 2	3.63	(1.11–11.8)	
***Area***	***Language region***	German	1.00	–	0.80
		French	0.81	(0.43–1.53)	
		Italian	1.02	(0.32–3.28)	
	***Urbanization***	Urban	1.00	–	0.71
		Peri-urban	0.82	(0.45–1.51)	
		Rural	1.03	(0.54–1.96)	

Hazard ratios adjusted for age from Cox regression models based on 2′515′961 women older than 18 years living in eligible households in Switzerland. *p* values from log-likelihood ratio tests. CI; confidence intervals.

## Discussion

The availability of information on all individuals living in Switzerland at the time of the census as well as on the composition of their households, combined with the linked mortality data, made it possible to identify homicide-suicide events that took place in households of two adults with or without children. We identified 73 such events, with a total of 158 deaths, during the period 1991–2008. The study showed that the perpetrators were predominantly men while the victims were women, confirming data from numerous case series [1], [14], [21], [22]. The age distributions were multimodal with peaks in middle and older ages, possibly reflecting events related to the dissolution of relationships (so-called ‘amorous jealousy’ [7]) and the burdens of caretaking (‘mercy killing suicide’ [3]), respectively. The incidence of homicide-suicides within households was similar in 1991 and 2001: around 3 events per million households and year.

The Swiss homicide rate of 0.9 per 100,000 is one of the lowest among European countries and has remained relatively stable for the last 20 years [23]. However, the percentage of homicides occurring within family settings, as well as the percentage of homicides followed by suicide, is higher in Switzerland than in other western nations [24]. About 60% of all homicides between 1980 and 2004 took place in private homes and 13% ended with the completed suicide of the perpetrator. This situation has been attributed to one of the highest percentages among European countries of households owning at least one firearm, i.e., approximately 28% [23]. A large proportion of these firearms are of military origin – the Swiss compulsory militia system distributes army weapons among soldiers. These weapons remain in private homes for as long as citizens serve in the army, and often beyond that time [4]. The prominent role of firearms and the high prevalence of gun ownership have also been observed in suicide deaths, especially among young males [25], [26]. Our study thus confirmed the leading role of firearms in the deaths of both victims and perpetrators. However, the role of military weapons could not be investigated because this information is not recorded in mortality statistics. Efforts are now underway to link criminological databases to the cohort data.

This is the first nation-wide cohort study of homicide-suicide. It allowed possible predictors of homicide-suicide events to be examined at the level of the individual, household and area: the Swiss censuses 1990 and 2000 included comprehensive data on demographic and socio-economic characteristic of residents, the composition of households and the geographical coordinates of buildings. Some of these variables were associated with the risk of committing a homicide-suicide act among men, or the risk of being killed in such an event in women. Among men, the risk was increased in divorcees and in foreigners without permanent residency in Switzerland. The risk was also increased in overcrowded households with two or more persons per room. Other studies have found that overcrowding increases the risk of violence, generally, and the risk of violence against women, in particular [27], [28].

These associations reflect situations and living conditions that are often associated with general psychological stress and intimate partner strain. Sociological and criminological theories have repeatedly linked psychological stress and life pressures with violence. In 1938 Merton put forward what has become known as ‘strain theory’: by putting strain on some members of society, social structures will make it more likely for some to commit crimes than for others [29]. More recently, Levin and Madfis [30] proposed a sequential model of cumulative strain to explain the origin of mass murder committed by students at their schools. The stages of the model, including chronic strain (for example due to long-standing family conflicts, jealousy or financial difficulties) and acute strain (for example following an argument, a break-up, a new partner) that initiates the planning of the violent act, may also be pertinent to homicide-suicide within families. Feminist theories address the fact that men predominate as perpetrators in homicide-suicide, an aspect neglected in many conceptual models of interpersonal violence [8]. They interpret homicide-suicide as a form of hegemonic masculinity, where the violent act is an extreme way of controlling female sexual partners and descendants and perceived as the only remaining option when relationships break down [8].

Interestingly, both in male perpetrators and female victims the risk was higher in individuals without religious affiliation, compared to Catholics. Women with no religious affiliation may on average be less amenable to authority structures (including patriarchy) and less bound by traditional relationship roles, than Catholic and Protestant women: the woman leaving the relationship is often the trigger for the escalation of violence [8]. Normative integration, where individuals accept the social norms and dogmas of a faith, may also play a role. The Catholic faith condemns murder, and condemns suicide as self-murder. According to the Catechism, “infanticide, fratricide, parricide, and the murder of a spouse are especially grave crimes by reason of the natural bonds which they break” [31]. Of note, in a recent analysis of the same cohort, we found that rates of suicide and assisted suicides were also substantially higher in those with no religious affiliation [18].

Factors unrelated to risk are also worth noting. In particular, current study found no evidence that homicide-suicide was associated with education or occupation, two variables that measure important components of socio-economic position (SEP) [32]. An article on episodes of homicide-suicide in Yorkshire and the Humber described the typical perpetrator as a “white male from the lower middle to working class” [8]. This may be a general misconception due to the shortcomings of much of the previous research in this area, which was generally based on case-series, without information on the population at risk. Alternatively, the epidemiology of homicide-suicide may differ in Switzerland. There was also no evidence for an association with nationality, the presence of children in the household, the language region and the degree of urbanization.

Homicide-suicides can be seen as essentially homicidal, with the killer subsequently dying by suicide, perhaps out of remorse. Alternatively, it can be seen as a suicide extended to intimate relations [33]. Studies comparing perpetrators of homicide-suicides with individuals committing murders or people dying by suicide showed that in comparison with simple murderers those committing homicides and then suicide were older [15], [34], more likely to be men [14], [34], [35], more likely to be married [15], [34], [35] and less likely to be unemployed [16], [34]. A history of domestic violence [35], and acting under the influence of alcohol [34], was common in perpetrators of homicide-suicides, but less common than in perpetrators of homicides. Finally, firearms were more likely to be used in homicide-suicide cases than in simple homicides [14], [16], [34], [36]. In Europe, among homicide-suicide events with multiple victims, 80% of victims were shot, compared to 38% in single homicides [37]. A history of attempted suicide was more common in individuals dying by simple suicide than in perpetrators of homicide-suicides [22]. Homicide-suicide events thus appear to represent a distinct entity, with characteristics distinguishing them both from homicides and suicides.

Our study has several limitations. Firstly, it relied on decennial census information for information about eligible households and their composition. The analysis thus excluded events that involved individuals not sharing the same household at the time of one of the two censuses, or events concerning individuals never registered as living together. Interestingly, the homicide-suicide offending rates observed in current study (0.09 and 0.10 per 100,000 in 1991 and 2001, respectively) are closely similar to the rate from a study of cases where an autopsy had been performed (0.09 per 100,000 for the period 1992–2004) [4], [38]. The latter rate will, however, also be too low because an autopsy is not always ordered. It is therefore likely that the true incidence of homicide-suicide events in Swiss households is slightly higher. Secondly, the study relied on data from death certificates and we therefore will have missed all cases of homicide followed by attempted suicide. Finally, it cannot be excluded that the analysis may have matched homicide and suicide events that were in fact unrelated. In other words, the person committing suicide may not have killed the members of his household. The very short time span between violent deaths, most of them by firearms and within the same household, makes this possibility highly unlikely. Finally, we did not investigate the role of household income or the role of the SEP of neighbourhoods. Data on household income are not collected in the census and the index of the SEP of neighbourhoods recently developed for Switzerland [39] was based on census 2000 data, whereas the present analysis is based on the 1990 census. We included education and occupation, which are important indicators of individual SEP, as well as crowding of households, which to some extent reflects the SEP of households.

In conclusion, this national longitudinal study in a country with wide-spread access to firearms shows that living conditions associated with psychological stress and lower levels of social support are associated with homicide-suicide events. Conversely, the study found no evidence that homicide-suicide was associated with education or occupation, nationality or the presence of children in the household. The linkage of criminological, forensic and psychological data with the national cohort study will overcome some of the limitations of the present study and allow more detailed characterisation of events, including the role of military and other firearms. Almost all incidents involved firearms and results thus add to a growing body of evidence that in Switzerland and elsewhere restricting access to firearms might prevent at least some homicide-suicide tragedies.
